# Flow visualization of an N95 respirator with and without an exhalation valve using schlieren imaging and light scattering

**DOI:** 10.1063/5.0031996

**Published:** 2020-11-01

**Authors:** Matthew Staymates

**Affiliations:** Material Measurement Laboratory, National Institute of Standards and Technology, 100 Bureau Drive, Gaithersburg, Maryland 20899, USA

## Abstract

This work demonstrates the qualitative fluid flow characteristics of a standard N95 respirator with and without an exhalation valve. Schlieren imaging was used to compare an adult male breathing through an N95 respirator with and without a valve. The schlieren imaging technique showed the flow of warm air passing through these respirators but did not provide information about droplet penetration. For this, strategic lighting of fog droplets was used with a mannequin head to visualize the penetration of droplets through both masks. The mannequin exhaled with a realistic flow rate and velocity that matched an adult male. The penetration of fog droplets was also visualized with a custom system that seals each respirator onto the end of a flow tube. Results of these qualitative experiments show that an N95 respirator without an exhalation valve is effective at blocking most droplets from penetrating through the mask material. Results also suggest that N95 respirators with exhalation valves are not appropriate as a source control strategy for reducing the proliferation of infectious diseases that spread via respiratory droplets.

As of the writing of this paper, the COVID-19 pandemic continues to disrupt normal life for most of the planet. The SARS-CoV-2 virus is currently understood to spread predominantly via respiratory droplets,^1–6^ as do many respiratory infectious diseases in humans.^7–10^ The U.S. Centers for Disease Control and Prevention (CDC) recommends the use of face coverings to help slow the spread of COVID-19.^11^ The literature shows the efficacy of masks and face coverings dating back to the 1918 influenza pandemic,^12,13^ and more recent work shows how masks and face coverings help reduce respiratory virus transmission.^14–17^ Multiple groups have now demonstrated that barrier face coverings and masks can help slow the spread of SARS-CoV-2.^18–23^ Of growing concern is the use of N95 respirators or face coverings that include an exhalation valve, as these are designed to allow exhaled air to pass through the mask unfiltered. Most exhalation valves consist of a small flexible tab that acts as a one-way check valve, opening upon exhalation and closing upon inspiration. The work presented here visually shows the differences between an N95 filtering facepiece respirator with and without an exhalation valve. This is demonstrated using fluid flow visualization. Different designs of N95 masks with valves may not all operate similarly. In this study, a model 8511 mask and a model 8210 mask (3M) were used.

Two fluid flow visualization techniques were used to qualitatively visualize the flow dynamics of an N95 filtering facepiece respirator with and without an exhalation valve. Here, we use schlieren imaging and light scattering of fog droplets to examine the filtering facepiece respirator, although others have recently used laser-based techniques.^22,24,25^ The schlieren optical technique has been previously applied to the study of infectious disease transport by observing the dynamics of human coughs, human thermal plumes, and flow patterns in indoor environments.^26–32^ Similarly, light scattering of droplets has been an effective tool for visualizing the droplet trajectory and travel distance.^33–35^

A single-mirror coincident schlieren optical system^36^ was used to visualize calm breathing of an adult male. An overview of this particular system, consisting of a 40 cm spherical mirror and accompanying optics, can be found in the literature^37,38^ and is also described in a public-service-announcement video released to the public.^39^ A schematic diagram of the optical system is provided in Fig. S1 of the supplementary material. Video data were collected with a high-speed camera (NOVA S9, Photron.com) at frame rates of 30 fps for human breathing and 125 fps for fog visualization.

To create repeatable and realistic exhalations, a mannequin head was modified to include 25 mm inner diameter flexible tubing from the mouth opening back through the head and exiting the back of the neck. The mouth opening was roughly an oval shape with a width of 25 mm and a height of 12.5 mm, modeled after measurements of the author’s anatomy during breathing. The flexible tubing was connected to a custom in-line fog generator, consisting of a Nichrome wire wrapped around a cotton plug. The cotton was soaked in an aqueous solution of 1% glycerin. Applying a power of 24 W (12 VDC/2 A) to the Nichrome wire generated a plume of fog droplets within the enclosed tube with a repeatable particle size distribution (see Fig. S2 of the supplementary material for droplet particle size distribution). A solid-state timer (model TMM-0999M, ametek.com) was used to pulse current into the fog generator for 2 s and provided a reasonably repeatable plug of fog droplets for each visualization experiment. The size distribution was measured with an Aerodynamic Particle Sizer (APS 3321, TSI.com) and is comparable to particle size distributions measured from humans during speaking, coughing, and exhaling.^40–42^

A custom exhalation system is located upstream of the fog generator and mannequin head. This system begins with an air compressor that feeds 550 kPa air to a shutoff valve and pressure regulator. Air is then fed into a 1.75 l accumulator at a preset pressure before an exhalation event. A solenoid valve (model VX2220, SMCpneumativs.com) and a second solid-state timer control the timing of the exhalation. When the solenoid is triggered, air exits the accumulator and then passes through a needle valve—this valve governs the flow rate of the exhalation. Air then enters a second 1.75 l accumulator, which helps widen the pulse width of the flow exiting the first accumulator and creates a flow profile that approaches that of a realistic human exhale (see Fig. S3 of the supplementary material).

Air exits the second accumulator and flows through flexible tubing into a straight-walled pipe with 2.06 cm inner diameter and 60 cm length. A high-intensity LED light (model F-55w, Xiamen Came Technology Co.) was strategically positioned behind the head of the mannequin to illuminate fog exiting the mouth. A hot wire anemometer (model FMA-905, Omega Engineering) was positioned at the end of the straight-walled pipe for velocity and flow rate measurements. The velocity or flow rate was monitored and adjusted in real-time with a custom LabVIEW data acquisition code (ni.com). Flexible tubing guided air to the fog generator and then exited the mannequin head. Triggering of the system was enabled with a manual control box that fires the solenoid valve and also triggers the image acquisition of the high-speed camera. An advantage of this system over a manual baffle-style pump or bicycle pump is the precise control over the air pressure, flow rate, pulse timing, and image acquisition, facilitating complete control over the expiratory flow rate and topography of this mannequin head and the image acquisition system. A schematic diagram of the overall setup can be found in Fig. S3 of the supplementary material, as well as a plot of the artificial flow profile compared to that of the author.

Another system was developed that helped in visualizing fog droplets penetrating these masks and consisted of a 50 mm straight-walled pipe and a custom mounting flange. The flange locked the fabrics or pieces of masks at the exit of the pipe and provided a seal with no gaps or leaks. This setup reduced the filtration surface area of the respirator by ∼75%, but it enabled a more controlled visualization experiment when compared to the mannequin system, which suffered some mask leakage around the chin, cheeks, and bridge of the nose. The same exhalation system described previously was used for this system. The face velocity of air upon the N95 respirators studied here was 34.5 cm/s and was based on maintaining the same flow rate between mannequin and pipe visualization experiments (peak flow of 42 l/min).

Figure 1 (multimedia view) shows two still images of the author breathing with two types of masks—an N95 respirator with (left) and without (right) an exhalation valve. The breathing frequency is synchronized in each case and repeats at a tempo of 100 beats/min, with inspiration for four clicks and then expiration for four clicks (using a metronome for consistency). These images show that the exhalation valve is operating exactly as designed. Respirators with valves are intended to decrease exhalation resistance and improve comfort to the wearer by readily dissipating humidity and heat from the dead space of the N95 respirator.^43^ However, as of the time of this writing, the CDC does not recommend the use of N95 respirators with valves because they do not filter droplets from exhaled air, which are believed to contribute to the spread of SARS-CoV2.^44^ The valved N95 respirator produces a turbulent jet that emanates from the valve during exhale and is vectored downwards from the wearer. In contrast, the standard N95 respirator shows the slow filtration of exhaled air transported through the filter during exhale. It is critically important to note that this fluid flow visualization technique does not show the transport of virus particles or droplets. Instead, it works by visualizing refractive index gradients (directly related to temperature and density in air), so this example is showing the warm air exiting the lungs and then moving through or out of the mask. Background disturbances that are visible around the neck are buoyant flows generated by the body heat escaping the volunteer.

**FIG. 1.**
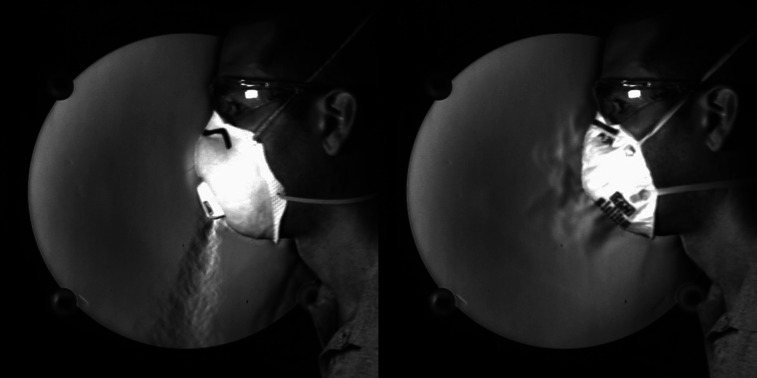
Schlieren images of an adult male exhaling in an N95 respirator with an exhalation valve (left) and without an exhalation valve (right). Multimedia view: https://doi.org/10.1063/5.0031996.110.1063/5.0031996.1

Figure 2 (multimedia view) shows the penetration of fog droplets through the N95 respirator with an exhalation valve. The face velocity of the flow at the exit is roughly 285 cm/s based on a flow rate of 42 l/min. The N95 respirator without an exhalation valve shows almost no fog penetration through the material. The images in Fig. 2 clearly demonstrate the reasoning behind the guidance from the CDC. The open exhale looks as though the mannequin is exhaling after a puff from a cigarette. The N95 respirator with the exhalation valve illustrates why this type of face respirator is not appropriate for infectious disease source control—the valve provides an easy path for droplets to exit the mask. Each of the still images shown here is timed together for the intercomparison of how the air jet behaves as it exits the mouth or respirator.

**FIG. 2.**
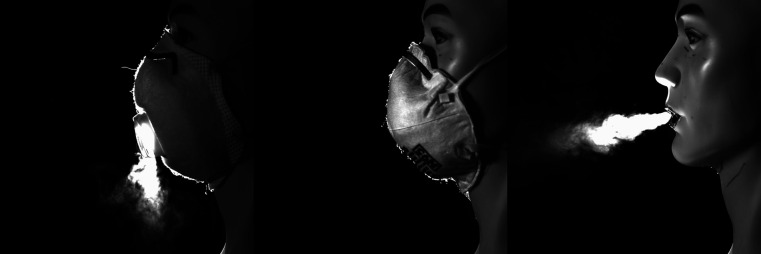
Still images extracted from the video footage of the mannequin exhaling with an exhalation valve (left), no exhalation valve (center), and no mask (right). The original video footage was captured at 125 FPS. Multimedia view: https://doi.org/10.1063/5.0031996.210.1063/5.0031996.2

Fog flow visualization of three examples (exhalation valve N95, regular N95, and no mask) is given in Fig. 3 (multimedia view). Here, each respirator style was cut from a new mask and fit into the mounting system (see the supplementary material for images of these masks mounted into the system). Droplet-laden flow was pushed at a flow rate that matched the mannequin (42 l/min); however, the face velocity was much lower (34.5 cm/s) because of the increased diameter of this pipe compared to the mannequin. Timing of this pulse was also extended because of a slight increase in the tubing length that led from the fog generator to the pipe system.

**FIG. 3.**
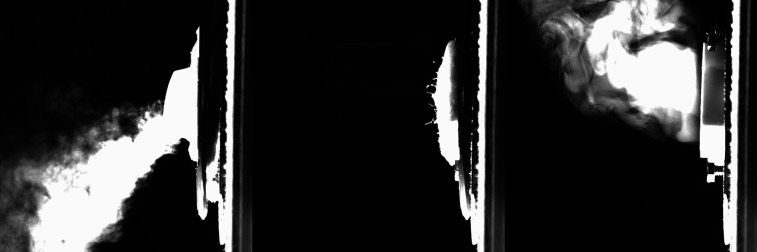
Pipe fog visualization showing an N95 respirator with an exhalation valve (left), N95 without an exhalation valve (center), and no mask (right). Flow is from right to left. The open pipe has a 51 mm diameter. Multimedia view: https://doi.org/10.1063/5.0031996.310.1063/5.0031996.3

Figure 3 (multimedia view) contains still images extracted from the same timestamp in each video. Just as in the mannequin example, here, we observe the exhalation valve allowing a very large number of droplets to exit the valve and not be collected by the N95 respirator material. The regular N95 respirator shows small streamlines of fog droplets emerging from the face of the material, but it is clear that this N95 respirator is capturing the majority of droplets.

N95 respirators with an exhalation valve produce a turbulent jet of exhaled air as the wearer exhales. This jet is directed downwards from the face and slows due to entrainment and mixing with the room air. Some fraction of exhaled air is filtered through the respirator material; however, the fog visualization experiments demonstrate that many droplets are transported through the valve and are not captured. Similar results were observed in a recent study.^24^ An N95 filtering facepiece respirator with an exhalation valve would not be an appropriate mitigation strategy for source control, as respiratory droplets from the wearer can easily pass through this valve and spread to others in close proximity.

The application of digital image processing of fluid flow visualization has been used in several studies to understand the dynamics of human sneezes, coughs, and droplet generation.^28,45–48^ Here, a simple image processing code was used to perform a semi-quantitative evaluation of the image data generated in Figs. 2 and 3 (Multimedia view). Written in LabVIEW, this code measures and sums the gray level intensities of each pixel of each frame within the video. This provides a running sum of overall pixel intensity that can be used as a quick assessment to understand trends between these three scenarios. Figure 4 shows results from processing three replicates of each example (open exhale/open pipe, N95, and exhalation valve N95) for both the mannequin and pipe fog visualization. When the pixel intensity is normalized to the “open” scenario, the exhalation valve N95 respirator shows a roughly 40% decrease in the pixel intensity for the mannequin and 25% decrease for the pipe. A decrease in the pixel intensity implies that some fraction of droplets are being captured by the mask and fewer droplets are penetrating through. The discrepancy between these two measurement techniques (mannequin vs pipe) is due to a difference in the total filtering area in each case. The valve size remains the same; however, the filtering material area on the pipe is roughly 85% less than that of the mannequin. The results suggest that the N95 respirator with an exhalation valve does provide some measurable reduction in droplet penetration through the respirator, possibly from droplet impaction on the valve components along with some fraction being filtered by the mask material. However, this type of N95 respirator would not be a viable option for source control of an infected person, as droplets can visually be seen emerging through the valve. The regular N95 respirator shows a 95% reduction in the pixel intensity. The low-level sinusoidal noise of the regular N95 respirator case of the mannequin is background illumination caused by fluorescent lights in the lab where these measurements were performed.

**FIG. 4.**
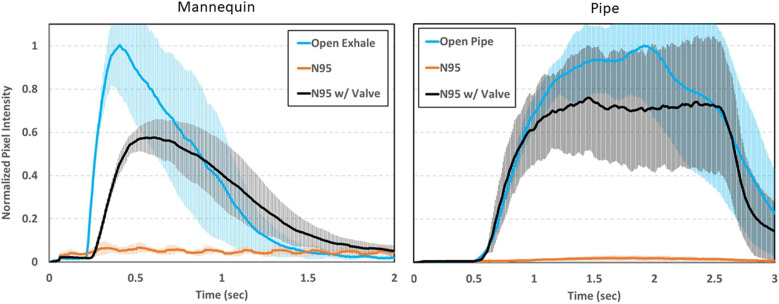
Image processing results of the mannequin and pipe fog visualization experiments. Pixel intensity values have been normalized to the maximum value in the “open” scenario. Error bars are the pointwise standard deviation from three replicates for each scenario.

These image processing calculations have limitations and should not be considered quantitative performance characteristics of an N95 respirator with and without an exhalation valve. This flow visualization measurement does not produce exactly the same number of droplets for each pulse, it does not illuminate all fog equally, and the three-dimensional nature of this flow precludes the camera’s ability to collect backscattered light from all droplets. The point here is to show that there is a clear trend between these three examples, and image processing can provide useful insights that help support the qualitative fluid flow visualization experiments.

In summary, fluid flow visualization techniques like schlieren imaging and backscattered fog illumination can be powerful tools to help understand the role fluid dynamics plays in the spread of infectious disease. This work helps illustrate the qualitative effectiveness and differences between two common filtering facepiece respirators. As with other recent work using fluid flow visualization techniques,^22,24^ the primary objective here is to create compelling visuals that are easy to understand and accessible to a broad audience. Additionally, this work may help with public awareness and perceptions about the usefulness of face coverings and masks.

See the supplementary material for information on the schlieren optical system setup, fog droplet particle size distribution, details of the artificial exhalation system, and flow profile of the mannequin.

## DATA AVAILABILITY

The data that support the findings of this study are available within the article and its supplementary material.
